# The New Jersey State Examining Board

**Published:** 1890-11

**Authors:** 


BUFFALO MEDICAL AND SURGICAL JOURNAL

A MONTHLY REVIEW OF MEDICINE AND SURGERY.

*EDITORS:*

THOMAS LOTHROP, M. D. - - WM. WARREN POTTER, M. D.

All communications, whether of a literary or business character, should be addressed to the editors : 284 FRANKLIN STREET, BUFFALO, N. Y.

VOL. XXX. NOVEMBER, 1890. No. 4.

### Editorial.

### THE NEW JERSEY STATE EXAMINING BOARD.

A RECENT exchange brings the news of the passage of the law establishing the State license for the practice of medicine in the adjacent commonwealth of New Jersey.

Dr. William Perry Watson, Secretary of the State Board of Medical Examiners for New Jersey, has kindly sent us a neat little reprint of the Act, which also contains the regulations for conducting the examinations under it.

We must confess to a little regret, as we read this new law, that New York's is not more like unto it, for we recognize some familiar features that were highly prized in the bill which our County Medical Society recommended to the State Legislature many years ago. Hereafter, there will be no question about the divorcement of the teaching and licensing powers in New Jersey. The diploma of the medical school is no longer a license to practise, but in order to make the divorce absolute and unconditional, the law provides that the members of the licensing board shall not be connected in any way with any medical college.

We well remember that the presence of a similar clause in the original Erie County bill brought to that measure many of its most vigorous enemies, and cost its friends frequent defeats ; but there were some of us that loved the clause for various reasons, among others for the enemies it made.

Another feature of this law calls for particular commendation, in that it expressly defines what is the practice of medicine. These examining-board laws are all essentially police regulations, having for their purpose and end the protection of the citizen from medical impropriety or incompetency. It is a weak spot in many of the acts that the practice of medicine is not defined, and the shrewd attorney has little difficulty in clearing his client from well-deserved penalty by the answer that his particular trick, abortion, feticide, tape-worm cure, bone-setting, mind cure, etc., etc., is not "practising" medicine. This stock answer will not work as well under the new law in Jersey. The greater efficiency of the measure as a police regulation is also secured by the clause giving the board authority to revoke license for certain causes.

This new member of the State licensing boards is of the "happy family" order,—physicians, homeopaths and eclectics are there in sweet accord and peace; and we trust that they may so unselfishly agree and that their work may so prosper, that, before the decade is over, their history may become a powerful object lesson to the legislators of the Empire State.

As State after State makes provision for giving citizens the advantages of the services of an educated and competent medical profession, the impetus of the movement becomes more and more manifest. In the near future, the charlatan, the quack, the medical ignoramus, will have trouble in deciding as to what community he will favor with his peculiar services.

It might prove an interesting item in statistics to ascertain the number of Canadian graduates who have thought it wiser to seek fame and fortune in the State of New York, rather than try the examination for license in their own country.

